# Wnt4 increases the thickness of the epidermis in burn wounds by activating canonical Wnt signalling and decreasing the cell junctions between epidermal cells

**DOI:** 10.1093/burnst/tkac053

**Published:** 2023-07-03

**Authors:** Fei Xiang, Pei Wang, Hao Gong, Jia Luo, Xin Zhou, Chenglin Zhan, Tianxing Hu, Mengru Wang, Yizhan Xing, Haiying Guo, Gaoxing Luo, Yuhong Li

**Affiliations:** Institute of Burn Research, State Key Laboratory of Trauma, Burns and Combined Injury, Southwest Hospital, Army Medical University, Chongqing 400038, PR China; Institute of Burn Research, State Key Laboratory of Trauma, Burns and Combined Injury, Southwest Hospital, Army Medical University, Chongqing 400038, PR China; Department of Cell Biology, Army Medical University, Chongqing 400038, PR China; Institute of Burn Research, State Key Laboratory of Trauma, Burns and Combined Injury, Southwest Hospital, Army Medical University, Chongqing 400038, PR China; Institute of Burn Research, State Key Laboratory of Trauma, Burns and Combined Injury, Southwest Hospital, Army Medical University, Chongqing 400038, PR China; Department of Cell Biology, Army Medical University, Chongqing 400038, PR China; Department of Cell Biology, Army Medical University, Chongqing 400038, PR China; Department of Cell Biology, Army Medical University, Chongqing 400038, PR China; Department of Cell Biology, Army Medical University, Chongqing 400038, PR China; Department of Cell Biology, Army Medical University, Chongqing 400038, PR China; Institute of Burn Research, State Key Laboratory of Trauma, Burns and Combined Injury, Southwest Hospital, Army Medical University, Chongqing 400038, PR China; Department of Cell Biology, Army Medical University, Chongqing 400038, PR China

**Keywords:** Wnt4, Wnt signalling pathway, Burn wound healing, Cell junction, Cell migration

## Abstract

**Background:**

Burn wound healing is a complex process and the role of Wnt ligands varies in this process. Whether and how Wnt4 functions in burn wound healing is not well understood. In this study, we aim to reveal the effects and potential mechanisms of Wnt4 in burn wound healing.

**Methods:**

First, the expression of Wnt4 during burn wound healing was determined by immunofluorescence, Western blotting and qPCR. Then, Wnt4 was overexpressed in burn wounds. The healing rate and healing quality were analysed by gross photography and haematoxyline and eosin staining. Collagen secretion was observed by Masson staining. Vessel formation and fibroblast distribution were observed by immunostaining. Next, Wnt4 was knocked down in HaCaT cells. The migration of HaCaT cells was analysed by scratch healing and transwell assays. Next, the expression of β-catenin was detected by Western blotting and immunofluorescence. The binding of Frizzled2 and Wnt4 was detected by coimmunoprecipitation and immunofluorescence. Finally, the molecular changes induced by Wnt4 were analysed by RNA sequencing, immunofluorescence, Western blotting and qPCR in HaCaT cells and burn wound healing tissues.

**Results:**

The expression of Wnt4 was enhanced in burn wound skin. Overexpression of Wnt4 in burn wound skin increased the thickness of epidermis. Collagen secretion, vessel formation and fibroblast distribution were not significantly impacted by Wnt4 overexpression. When Wnt4 was knocked down in HaCaT cells, the ratio of proliferating cells decreased, the ratio of apoptotic cells increased and the ratio of the healing area in the scratch healing assay to the number of migrated cells in the transwell assay decreased. The nuclear translocation of β-catenin decreased in shRNA of Wnt4 mediated by lentivirus-treated HaCaT cells and increased in Wnt4-overexpressing epidermal cells. RNA-sequencing analysis revealed that cell junction-related signalling pathways were significantly impacted by Wnt4 knockdown. The expression of the cell junction proteins was decreased by the overexpression of Wnt4.

**Conclusions:**

Wnt4 promoted the migration of epidermal cells. Overexpression of Wnt4 increased the thickness of the burn wound. A potential mechanism for this effect is that Wnt4 binds with Frizzled2 and increases the nuclear translocation of β-catenin, thus activating the canonical Wnt signalling pathway and decreasing the cell junction between epidermal cells.

Highlights This study systematically researches the role of Wnt4 in burn wound healing.

- Wnt4 promotes the migration of epidermal cells.
- Wnt4 binds with Frizzled2 and activates the canonical Wnt signaling pathway.
- Wnt4 decreases the cell junction between epidermal cells.

## Background

As the largest organ of the body, skin is composed of the epidermis, dermis and hypodermis layers. The skin is prone to damage by physical, chemical and biological factors. Burn wounds are one of the main causes of skin damage. Burn wound healing is a complex process. Many factors, such as burn depth, infection, nutrition, ageing and oxygen, can impact the quality of healed skin [1]. In terms of cell level, the amount and quality of epidermal cells is one of the determining factors that impact the prognosis of burn wounds. Epidermal cells may migrate from the adjacent area or differentiate from migrated stem cells. Many signalling pathways have been reported to be involved in this process, including Wnt, Notch and transforming growth factor beta [2–4].

The Wnt protein family is a series of highly conserved secretory glycoproteins that play roles through autocrine or paracrine signalling. Wnt signalling participates in the healing of mucosal tissue and airway epithelium [5–7] and the activation of the canonical Wnt signalling pathway has been reported to be invaluable for the healing of skin wounds [8]. Several Wnt ligands have been reported to play roles in the wound healing or regeneration of skin and its appendages. For example, Wnt7a and Wnt7b are involved in hair follicle regeneration after wounding [9]. Wnt10b overexpression induces hair follicle regeneration [10,11]. Exosome-mediated Wnt4 signalling has been reported to be required for burn wound healing [12], which suggests that Wnt4 may be a potential new target in treating wound healing. However, further research is required to reveal the exact role and mechanism of Wnt4 in burn wound healing. In this study, we found that Wnt4 was a positive factor for burn wound healing. Through *in vitro* and *in vivo* experiments, we also found that Wnt4 may decrease cell junctions and promote the migration of epidermal cells.

## Methods

### Animals and vectors

Male Kunming mice were obtained from the Laboratory Animal Center of the Army Medical University, Chongqing, China. All animal-related procedures were conducted in strict accordance with the approved institutional animal care and maintenance protocols. All experimental protocols were approved by the Laboratory Animal Welfare and Ethics Committee of the Army Medical University (ethics approval No. AMUWEC20210247). The shRNA of Wnt4 mediated by lentivirus (shWnt4) and the the empty vector for shWnt4 (shGFP, where GFP is green fluorescent protein) vectors were constructed by Tsingke Biotechnology Co., Ltd (Beijing, China). The adenovirus-mediated overexpression of mouse Wnt4 (AdWnt4) and the empty vector for AdWnt4 (AdGFP) vectors were constructed by Obio Technology Corp., Ltd (Shanghai, China).

### Cell culture

The culture medium for HaCaT cells consisted of RPMI 1640 and 10% foetal bovine serum. Cells were cultured at 37°C with 5% CO_2_ in a cell culture incubator. For lentivirus infection, 2 × 10^5^ cells were seeded onto 6-well plates, 36 μl of lentivirus (titre 1.1 × 10^8^) was added to the wells on the second day and the culture medium was changed on the third day. Three days later, the cells were passaged and a final concentration of 2 μg/ml puromycin (Beyotime, China) was added to the culture medium to obtain stable cell lines.

### Burn model

The mice were anaesthetized with isoflurane. A piece of metal 2 cm in diameter was heated to 120°C. Full thickness skin burns were made with the heated metal for 15 s. Then, 100 μl of AdWnt4 or AdGFP (titre 1.1 × 10^9^) was injected intradermally into the edge of the burns. At 7, 14 and 21 days after injection, the mice were sacrificed and burns with edges were collected. The samples were gradually dehydrated and embedded in paraffin. They were then cut into 5 μm sections and gradually hydrated before haematoxyline and eosin (H&E) staining, Masson staining or immunofluorescence staining.

### Immunofluorescence

First, the hydrated sections or coverslips with cells were blocked with blocking reagent (Beyotime, China) for 1 h. Then, they were incubated with primary antibodies overnight. Primary antibodies against the following proteins were used: Wnt4 (diluted 1:200, Abcam, USA), E-cadherin, integrin subunit alpha 6 (ITGα-6), integrin subunit beta 1 (ITGβ-1), Frizzled2 (all diluted 1:100, Bioss, China), ZO-1, occludin, claudin-1 (all diluted 1:100, Proteintech, China) and GFP and β-catenin (all diluted 1:100, Santa Cruz, USA). After that, the samples were incubated with Cy3-labelled or Cy5-labelled secondary antibodies (diluted 1:500, Beyotime, China) for 1 h. The sections were then counterstained with 4′,6-diamidino-2-phenylindole (DAPI; Beyotime, China). Finally, they were mounted with antifade mounting medium (Beyotime, China) and observed under a microscope.

### H&E staining

H&E staining was performed as previously described [13]. Briefly, the hydrated sections were stained with haematoxylin (Zhongshan Goldenbridge, China) for 2 min and subsequently rinsed with water. The sections were later stained with eosin (Zhongshan Goldenbridge, China) for 2 min and rinsed with water thereafter. After gradual dehydration, the sections were mounted with neutral gum (Zhongshan Goldenbridge, China) and observed under a microscope.

### Masson staining

A modified Masson’s trichrome stain kit (Solarbio, China) was used to perform the staining. The procedures were performed according to the manufacturer’s instructions. Briefly, slides were incubated in mordant dye overnight. After washing with water, the slides were stained with Azurol blue and washed. Then, Mayer haematoxylin was used to stain for 2 min and washed. Before staining with fuchsia, the slides were differentiated with acidic differentiation solution. After these steps, phosphomolybdic acid and aniline blue were separately used to stain the slides. Finally, the slides were washed with a weak acid solution, gradually dehydrated and mounted with neutral gum (Zhongshan Goldenbridge, China).

### Western blotting

Cells were lysed with RIPA buffer (Beyotime, China). A tris-glycine extended stain-free FastCast acrylamide kit (10%, Bio-Rad, USA) was used to prepare the PAGE gels. Proteins (20 μg) were loaded to run Sodium Dodecyl Sulfate PolyAcrylamide Gel Electrophoresis (SDS-PAGE) and transferred to polyvinylidene difluoride membranes. The membranes were subsequently blocked with 5% nonfat milk in Tris-buffered saline buffer containing 0.1% Tween-20 and then incubated with primary antibodies at 4°C overnight. The following primary antibodies were used: β-catenin (diluted 1:20,000; Santa Cruz, USA), p-β-catenin (diluted 1:5000; CST, USA), Wnt4 (diluted 1:20,000; Abcam, USA), E-cadherin, ITGα6, ITGβ1, Frizzled2 (all diluted 1:10,000, Bioss, China), ZO-1, occludin, claudin-1 (all diluted 1:10,000, Proteintech, China) and β-actin (diluted 1:8000, Sigma, USA). This was followed by incubation with horseradish peroxidase-conjugated secondary antibody (diluted 1:10,000; Beyotime, China) for 1 h. Then, signals were detected using enhanced chemiluminescent substrates (Beyotime, China). Images were acquired and quantified using Image Plus (Bio-Rad, USA).

### Reverse transcription polymerase chain reaction (RT-PCR) assay

The total RNA of cells or tissues was extracted using TRIzol (Invitrogen, USA) following the manufacturer’s instructions. cDNA was synthesized using an RT-PCR kit (Toyobo, Japan). A Supermix kit (Bio-Rad, USA) was used to amplify the cDNA. The primers used are summarized in supplementary Table S1, see online supplementary material.

### C‌CK-8 assay

A total of 2000 cells per well were seeded onto 96-well plates. Wnt4 protein (Proteintech, China) was added to a final concentration of 1 ng/μl the next day. shWnt4 or shGFP was added to a final titre of 10^6^ plaque forming unit per ml. The optical density (OD) value was detected and analysed according to the protocol recommended by the CCK-8 kit manufacturer (Beyotime, China).

### Fluorescence activated cell sorting (FACS) assay

The experiments were performed according to the protocol recommended by the kit manufacturer. For cell cycle detection, a Cell Metre™ Fluorimetric live cell cycle assay kit (AAT Bioquest, USA) and cell cycle staining kit (Multi Science, China) were used. For cell apoptosis, a Pacific Blue™ annexin V apoptosis detection kit with PI (Biolegend, USA) was used.

### Scratch healing assay

HaCaT cells were infected with shWnt4 or GFP vector 1 day before use. A total of 6.0 × 10^5^ cells per well were seeded onto 6-well plates. PNU-74654 (MCE, China) was added to the cells at a final concentration of 100 μM at 6 h after seeding. Scratches were made with a 10 μl pipette at the same time. The scratches were photographed at 0, 3, 6 and 24 h after treatment. The ratio of the healing area to wound area of the scratches was calculated with ImageJ software.

### Transwell assay

Transwells (8.0 μm, Jet Biofiltration, China) were placed into 24-well plates. A total of 1.0 × 10^5^ cells per well were seeded into the wells. RPMI 1640 culture medium with 20% foetal bovine serum was used to induce the migration of cells. The cells were stained with crystal violet (Beyotime, China) 24 h after seeding and counted with ImageJ software.

### RNA sequencing

HaCaT cells were infected with shGFP or shWnt4. After 48 h, the cells were collected and sent to Tsingke Biotechnology Co., Ltd (Beijing, China) for RNA sequencing. Three independent samples were used for each group. Differential expression analysis was performed using the DESeq2 R package. The false discovery rate (FDR) was obtained through Benjamini–Hochberg correction. Genes with FDR < 0.01 and |log2(foldchange)| ≥ 2 found by DESeq2 were assigned as differentially expressed. KOBAS software was used to test the statistical enrichment of differentially expressed genes in KEGG pathways.

### Immunoprecipitation assay

Immunoprecipitation assays were performed with a Co-IP kit (Beyotime, China). According to the kit instructions, RIPA buffer was used to lyse the cells to extract the protein. After measuring the protein concentration, 400 μg of the protein sample was added to 1 mg of Wnt4 (Abcam, USA) or Frizzled2 (Bioss, China) antibody, and the lysate was adjusted to a volume of 500 μl and shaken at 4°C overnight. The immunoprecipitates were eluted and used for Western blot analysis.

### Statistical analysis

ImageJ 1.4 (NIH, USA) was used to measure the area of burn wounds. Image-Pro Plus 6 (MediaCybernetics, USA) was used to measure the intensity of bands and the thickness of the epidermis. GraphPad Prism 8 (Dotmatics, USA) was used for statistical analysis. An unpaired t test was used to compare the significant difference between two groups. Ordinary one-way ANOVA with multiple comparisons was used to compare the significant difference among three or more groups. Two-way ANOVA with multiple comparisons was used to compare the significant difference in data with both time and treatment factors. *P* < 0.05 was considered significant. Three independent experiments were performed for each group. To observe the ratio of healing area to burn wound area, six mice were used for every time point in each group.

## Results

### Wnt4 increases the thickness of epidermis in burn wounds

**Figure 1:**
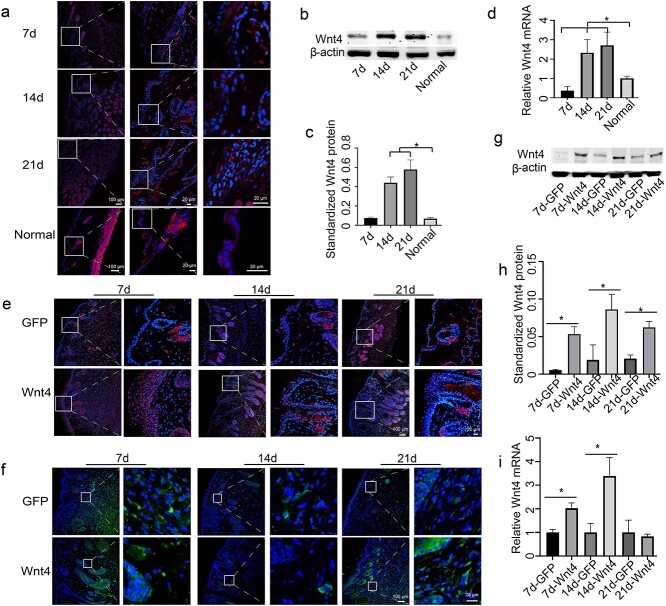
Expression and overexpression of Wnt4 in burn wound healing skin. (**a**) The expression of Wnt4 in the burn wound edge was detected by immunofluorescence. Normal skin with no burns was used as a control. DAPI was used to counterstain the nucleus. The expression in sebaceous glands and muscle is nonspecific. The middle panels are an enlargement of the framed areas in the left panels. The right panels are an enlargement of the framed areas in the middle panels. Normal, skins with no burns. The scale bars for the upper three panels are the same. Scale bar for the left panels is 100 μm. Scale bar for the middle and right panels is 20 μm. (**b**, **c**) Western blot analysis of the expression of Wnt4 in burn wounds. β-Actin was used as an internal control. (**d**) qPCR analysis of the expression of Wnt4 in burn wounds. (**e**) Immunofluorescence analysis of the expression of Wnt4 in AdGFP- or AdWnt4-treated burn wounds. (**f**) Immunofluorescence analysis of the expression of GFP in AdGFP- or AdWnt4-treated burn wounds. (e, f) DAPI was used to counterstain the nucleus. The right panels are an enlargement of the framed areas in the left panels. Scale bar for the left panels is 100 μm. Scale bar for the right panels is 20 μm. (**g**) Western blot analysis of the expression of Wnt4 in AdGFP- or AdWnt4-treated burn wounds. β-Actin was used as an internal control; (**h**) is the statistical analysis of (g). (**i**) qPCR analysis of the expression of Wnt4 in AdGFP- or AdWnt4-treated burn wounds. ^*^p < 0.05; *n* = 3. *DAPI* 4′,6-Diamidino-2-phenylindole, *GFP* green fluorescent protein, *AdWnt4* adenovirus-mediated overexpression of mouse Wnt4, *AdGFP* the empty vector for AdWnt4

**Figure 2:**
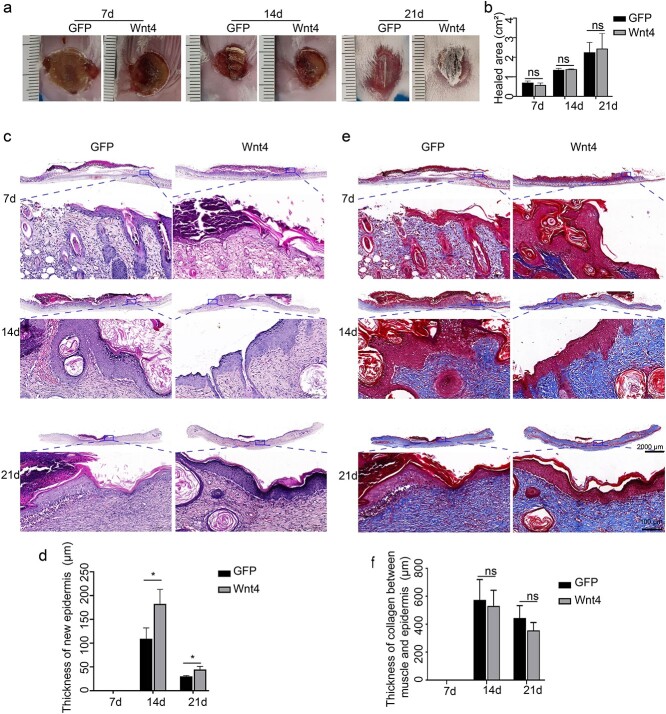
Effect of Wnt4 on burn wound healing. Burns 2 cm in diameter were made on 8-week- old mice and AdWnt4 or AdGFP was intradermally injected into the edge of the burns. Mice were sacrificed at 7 (7d), 14 (14d) or 21 days (21d) after injection. (**a**) The burn wound was photographed. (**b**) The area of the healed wound was analysed; *n* = 6. (**c**) The burn wound was observed after H&E staining. (**d**) Statistical analysis of the thickness of the epidermis in (c); *n* = 3. (**e**) The burn wound was observed after Masson staining. (c, e) The lower panel is the enlargement of the framed area in the upper panel. Scale bar for the upper panel is 2000 μm. Scale bar for the lower panel is 100 μm. (**f**) Statistical analysis of the thickness of collagen between the muscle and the epidermis in (e); *n* = 3. ^*^p < 0.05; ns, no significant difference. *GFP* green fluorescent protein, *AdWnt4* adenovirus-mediated overexpression of mouse Wnt4, *AdGFP* the empty vector for AdWnt4

**Figure 3:**
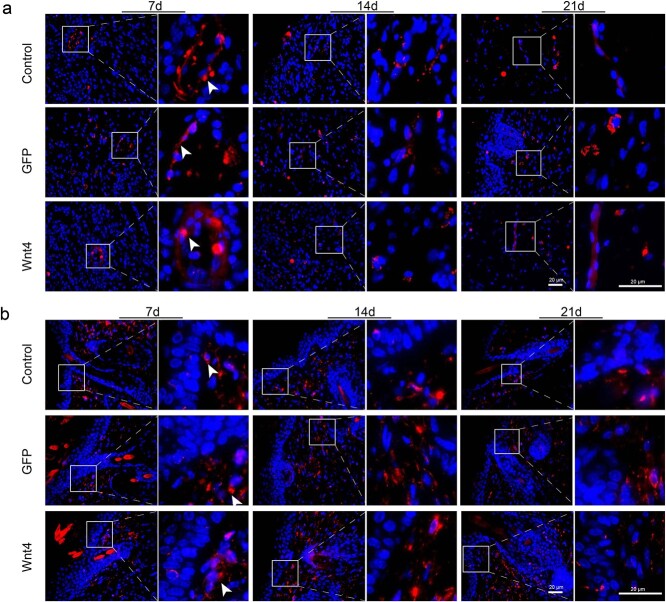
Expression of CD31 and vimentin in AdWnt4-treated burn wound edges. (**a**) Immunofluorescence analysis of the expression of CD31 in the burn wound edges. (**b**) Immunofluorescence analysis of the expression of Vimentin in the burn wound edges. The right panels are an enlargement of the framed areas in the left panels; scale bar = 20 μm. *DAPI* 4′,6-Diamidino-2-phenylindole, *GFP* green fluorescent protein, *AdWnt4* adenovirus-mediated overexpression of mouse Wnt4, *AdGFP* the empty vector for AdWnt4, *Control* burn wound with no treatment

First, the expression of Wnt4 in the burn model was tested by immunofluorescence. Wnt4 was expressed in the cytoplasm of epidermal cells. Compared with normal unwounded skin, the expression of Wnt4 in burn wound skin was enhanced in both hair follicle cells and epidermal cells (Figure 1a–d). To determine the role of Wnt4 in burn wound healing, AdWnt4 was intradermally injected into the edge of the burn and the healing process was monitored by both gross observation and microscopy. Immunofluorescence staining of GFP showed that both AdWnt4 and AdGFP were injected and expressed in the injection site (Figure 1e). Immunofluorescence staining of Wnt4 further showed that Wnt4 was overexpressed successfully in the injection area (Figure 1f). Compared to the AdGFP-treated group, the area of healed wounds in the AdWnt4-treated group was not significantly different (Figure 2a, b). However, when the burned area was sectioned and observed after H&E staining, it was obvious that the epidermis in the AdWnt4-treated group was thicker than that in the AdGFP-treated group. The thickness of the epidermis began to thin 21 days post-injection (Figure 2c, d). This implies that the healing quality of burns may be better in the AdWnt4-treated group. Masson staining showed that the distribution of collagen was not significantly different between the AdWnt4 group and the AdGFP group (Figure 2e). Immunostaining for CD31 and vimentin showed that the angiogenesis and fibroblast distribution was not significantly different between the AdWnt4 group and the AdGFP group (Figure 3).

### Knockdown of Wnt4 inhibits the migration of HaCaT cells

**Figure 4:**
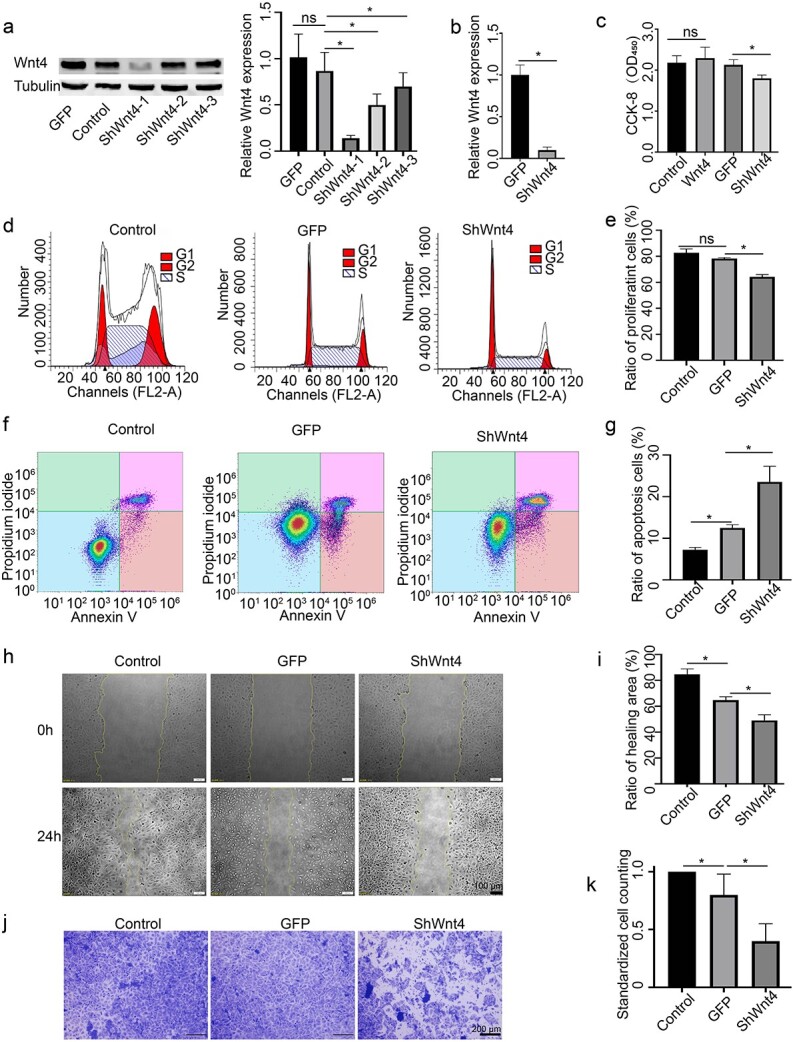
Effects of Wnt4 on HaCaT cells. HaCaT cells were infected with shWnt4 or shGFP lentivirus. (**a**) Western blot analysis of the expression of Wnt4. Tubulin was used as an internal control. The right panel histogram shows the statistics of the left panel data. (**b**) qPCR analysis of the expression of Wnt4. (**c**) The proliferation of shWnt4-treated HaCaT cells was analysed using a CCK-8 assay. (**d**) FACS analysis of the proliferation of shWnt4-treated HaCaT cells; (**e**) the statistics of (d). (**f**) FACS analysis of apoptosis of shWnt4-treated HaCaT cells; (**g**) shows the statistics of (f). (**h**) The migration of shWnt4- treated HaCaT cells was analysed using a scratch assay; (**i**) shows the statistics of (h). Scale bar = 100 μm. (**j**) The migration of shWnt4-treated HaCaT cells was analysed using a transwell assay (scale bar = 200 μm); (**k**) shows the statistics of (j). *n* = 3, ^*^p < 0.05, ns, no significant difference. *Control* cells with no treatment, *GFP* green fluorescent protein, *ShWnt4* shRNA of Wnt4 mediated by lentivirus, *ShGFP* the empty vector for shWnt4

To determine the potential mechanism by which Wnt4 regulates the activities of epidermal cells, three shRNAs targeting Wnt4 were constructed and transduced into HaCaT cells. While all three shRNAs were successful, shWnt4–1 had the best knockdown efficiency (Figure 4a, b). Therefore, we subsequently refer to shWnt4–1 as shWnt4. Wnt4 protein was also added to the culture medium of HaCaT cells. However, the CCK-8 assay showed that the addition of Wnt4 protein did not obviously change the proliferation of HaCaT cells, whereas shWnt4 inhibited the proliferation of HaCaT cells (Figure 4c). Thus, only shWnt4 was used to test the role of Wnt4 in the subsequent *in vitro* experiments. The cell cycle and cell apoptosis of shWnt4-treated HaCaT cells were analysed using FACS. Compared to that in the AdGFP group, the ratio of proliferating cells in the shWnt4 group was lower (Figure 4d, e), while the ratio of apoptotic cells in the shWnt4 group was higher (Figure 4f, g). The wound scratch assay revealed that at 24 h after treatment, the ratio of healing area in the shWnt4 group was lower than that in the control group or AdGFP group (Figure 4h, i). Transwell assays also demonstrated that shWnt4 inhibited the migration of HaCaT cells (Figure 4j, k).

### Wnt4 activates Wnt/β-catenin signalling in burn wound healing

**Figure 5:**
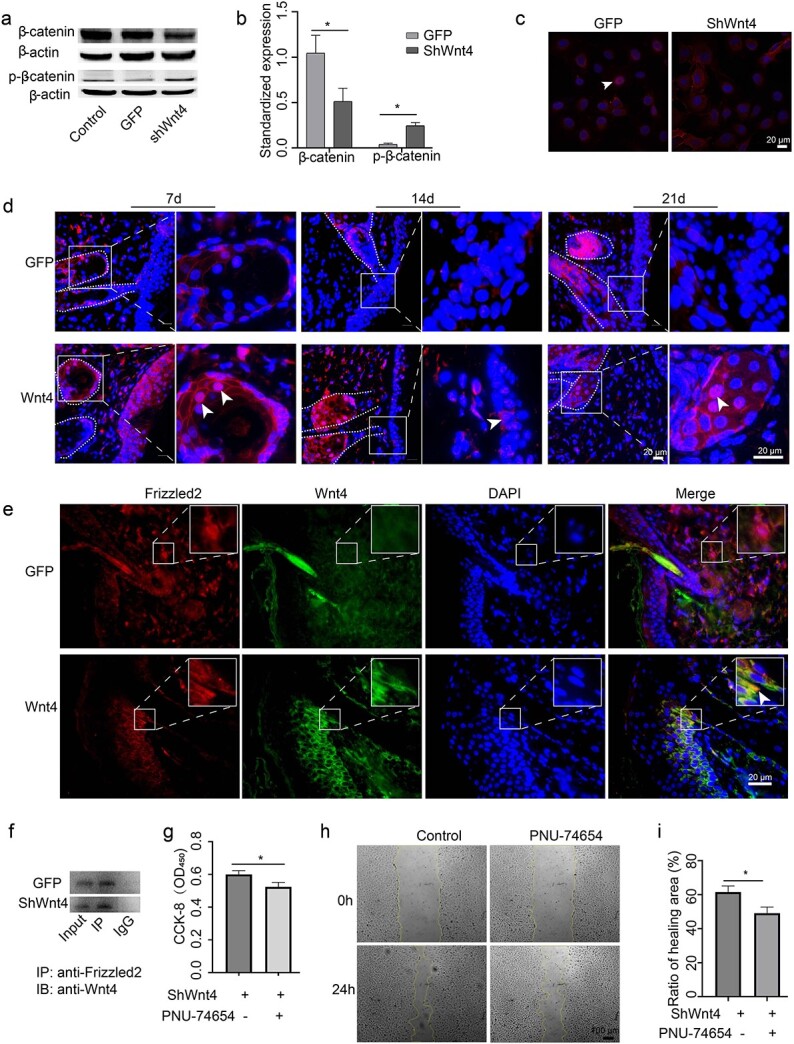
Wnt4 activates the canonical Wnt signalling pathway. (**a**–**c**) HaCaT cells were infected with shWnt4 or GFP lentivirus. (a) Western blot analysis of the expression of β-catenin and p-β-catenin; β-actin was used as an internal control; (b) shows the statistics of (a). (c) Immunofluorescence analysis of the expression of β-catenin. DAPI was used to counterstain the nucleus. An arrowhead indicates the nuclear translocation of β-catenin. (**d**) Immunofluorescence analysis of the expression of β-catenin in AdWnt4- treated burn wound edges. DAPI was used to counterstain the nucleus. Arrowheads indicate the nuclear translocation of β-catenin. The right panels are an enlargement of the framed areas in the left panels. (**e**) Immunofluorescence analysis of the expression of Frizzled2 and Wnt4 in AdWnt4-treated burn wound edges. DAPI was used to counterstain the nucleus. An arrowhead indicates the coexpression of Frizzled2 and Wnt4. The upper right inserted area is the enlargement of the framed area in the picture. (**f**) Coimmunoprecipitation analysis of the coexpression of Frizzled2 and Wnt4 in shWnt4-treated HaCaT cells. (**g**) The proliferation of PNU-74654- and shWnt4-treated HaCaT cells was analysed using a CCK-8 assay. (**h**) The migration of PNU-74654- and shWnt4-treated HaCaT cells was analysed using a scratch assay; (**i**) shows the statistics of (h). Scale bar = 20 μm; *n* = 3, ^*^p < 0.05. *Control* cells with no treatment, *DAPI* 4′,6-diamidino-2-phenylindole, *GFP* green fluorescent protein, *ShWnt4* shRNA of Wnt4 mediated by lentivirus, *ShGFP* the empty vector for shWnt4, *AdWnt4* adenovirus-mediated overexpression of mouse Wnt4, *AdGFP* the empty vector for AdWnt4. *IP *immunoprecipitation, *IB* immunoblotting

The relative expression of β-catenin in the shWnt4 group was lower than that in the GFP group, whereas the relative expression of p-β-catenin in the shWnt4 group was higher than that in the GFP group (Figure 5a, b). The nuclear translocation of β-catenin was also inhibited by shWnt4 (Figure 5c). We also examined the expression pattern of β-catenin in AdWnt4-treated burn wound skin. From 7 days post-injection to 21 days post-injection, both the expression and nuclear translocation of β-catenin were enhanced in the AdWnt4-treated group (Figure 5d). Immunostaining of Wnt4 and Frizzled2 together showed that they were coexpressed in some epithelial cells at the edge of burn wound skin (Figure 5e). Their coexpression was further validated by co-IP in HaCaT cells (Figure 5f). PNU-74654, a known inhibitor of Wnt/β-catenin signalling, was also used to treat shWnt4-treated HaCaT cells. The CCK-8 assay showed that after PNU-74654 treatment, the proliferation of the cells was further inhibited (Figure 5g). Scratch healing assays revealed that the migration of the cells was further inhibited by PNU-74654 (Figure 5h, i). These data demonstrate that Wnt4 binds with Frizzled2, increases the expression of β-catenin and activates the Wnt/β-catenin signalling pathway to regulate the activities of epidermal cells.

### Wnt4 impacts the cell junction between epidermal cells *in vitro* and *in vivo*

**Figure 6:**
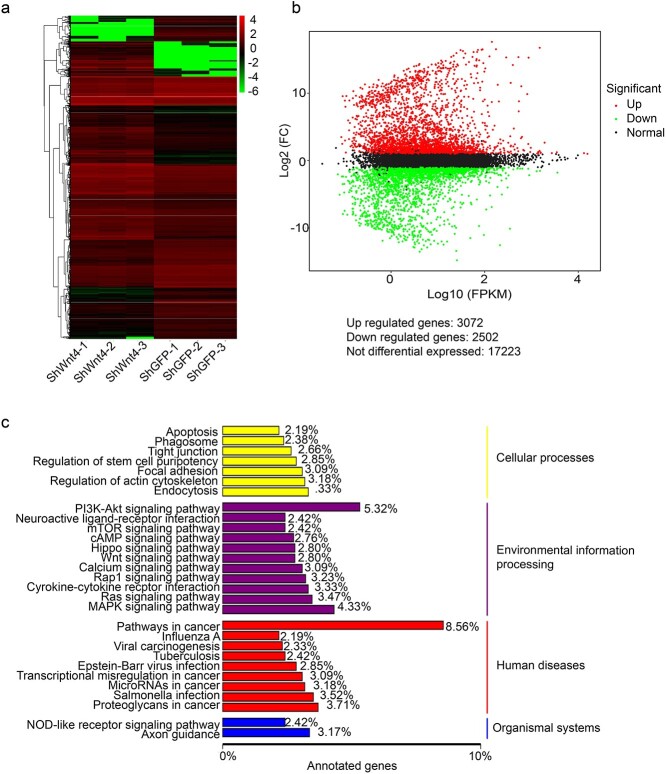
RNA sequence analysis of shWnt4-treated HaCaT cells. (**a**) Cluster analysis of the differentially expressed genes. (**b**) M *vs* A plot of the differentially expressed genes. (**c**) Pathway type cluster in KEGG analysis of the differentially expressed genes. *n* = 3. *ShWnt4* shRNA of Wnt4 mediated by lentivirus, *ShGFP* the empty vector for shWnt4

We next examined how Wnt4 impacts the proliferation and migration of epidermal cells. RNA sequencing was used to screen the potential mechanisms. After shWnt4 treatment, 3072 genes were upregulated, while 2502 genes were downregulated (Figure 6a, b). KEGG enrichment analysis showed that cell junction and cell adhesion were among the top impacted cellular processes (Figure 6c). Since the migration of HaCaT cells was significantly impacted by shWnt4, and cell junctions are a key factor for cell migration, we focused on the cell junction change induced by Wnt4.

**Figure 7:**
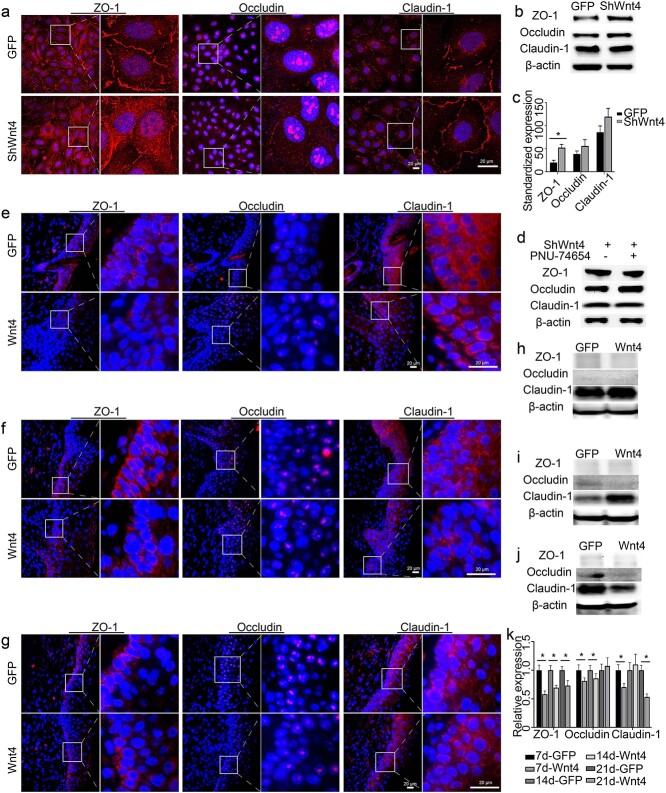
Effect of Wnt4 on some tight junction proteins between epidermal cells. The expression levels of ZO-1, occludin, and claudin-1 were detected by immunofluorescence (**a**, **e**, **f**, **g**), Western blot (**b**–**d**, **h**–**j**) and qPCR (**k**). (a, e, f, g) The right panel is an enlargement of the framed area in the left panel. DAPI was used to counterstain the nucleus. (a–d) The expression of the three proteins in shWnt4- or PNU-74654- treated HaCaT cells; (c) shows the statistics of (b). (e–k) A 2 cm diameter burn was made in 8-week-old mice and AdWnt4 or AdGFP was intradermally injected into the edge of the burns. Mice were sacrificed at 7 days (e, h, k), 14 days (f, i, k), or 21 days (g, j, k) after injection. Scale bar = 20 μm; *n* = 3, ^*^p < 0.05. *DAPI* 4′,6-Diamidino-2-phenylindole, *ZO- 1* tight junction protein 1, *GFP* green fluorescent protein, *ShWnt4* shRNA of Wnt4 mediated by lentivirus, *ShGFP* the empty vector for shWnt4, *AdWnt4* adenovirus-mediated overexpression of mouse Wnt4, *AdGFP* the empty vector for AdWnt4

The expression of ZO-1, occludin, and claudin-1 was detected by immunofluorescence and western blotting in both cultured HaCaT cells *in vitro* and skin *in vivo*. In HaCaT cells, ZO-1 and claudin were mostly expressed at the edge of the cells, while occludin was mostly expressed in the nucleus. When Wnt4 was knocked down, the expression of ZO-1 was increased at the edge of the cells, whereas the expression of occludin and claudin-1 was not obviously changed (Figure 7a). The western blot results were consistent with the immunofluorescence results (Figure 7b, c). When PNU-74654 was added to the culture medium, the expression of occludin was further increased (Figure 7d). In the epidermis, ZO-1 and claudin-1 were mostly expressed at the edge of the cells, while occludin was mostly expressed in the nucleus (Figure 7e–g). At 7 days after Wnt4 overexpression, the expression levels of the three proteins were not changed in the burn wound (Figure 7h). At 14 days after Wnt4 overexpression, the expression of ZO-1 was not changed. The expression of occludin decreased, whereas the expression of claudin-1 increased in the burn wounds (Figure 7i). At 21 days after Wnt4 overexpression, the expression levels of the three proteins all decreased in the burn wound (Figure 7j). The mRNA expression levels of the three proteins in burn wounds were consistent with the protein expression levels (Figure 7k).

**Figure 8:**
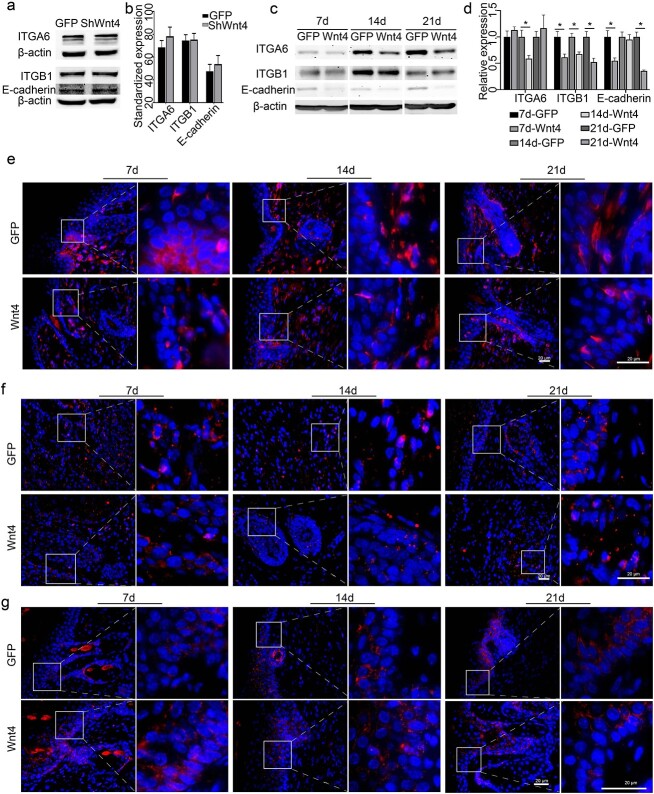
Effect of Wnt4 on some cell adhesion molecules between epidermal cells. The expression levels of ITGα6, ITGβ1 and E-cadherin were detected by western blotting (**a**–**c**), qPCR (**d**) and immunofluorescence (**e**–**g**). (a, b) The expression of the three proteins in shWnt4-treated HaCaT cells; (b) shows the statistics of (a). (e–g) A 2 cm diameter burn was made in 8-week-old mice and AdWnt4 or AdGFP was intradermally injected into the edge of the burns. Mice were sacrificed at 7, 14 or 21 days after injection. The right panels are an enlargement of the framed areas in the left panels. DAPI was used to counterstain the nucleus. (e) The expression of ITGα6. (f) The expression of ITGβ1. (g) The expression of E- cadherin. Scale bar = 20 μm; *n* = 3, ^*^p < 0.05. *ITGα6* Integrin subunit alpha 6, *ITGβ1* integrin subunit beta 1, *DAPI* 4′,6-diamidino-2- phenylindole, *GFP* green fluorescent protein, *ShWnt4* shRNA of Wnt4 mediated by lentivirus, *ShGFP* the empty vector for shWnt4, *AdWnt4* adenovirus-mediated overexpression of mouse Wnt4, *AdGFP* the empty vector for AdWnt4

ITGα6, ITGβ1 and E-cadherin are important known cell adhesion molecules that play a part in cell junctions in the epidermis. Therefore, we also tested the expression pattern of these three proteins in shWnt4-treated HaCaT cells and AdWnt4-treated burn wound skin. When Wnt4 was knocked down in HaCaT cells, the expression levels of the three molecules were not changed (Figure 8a, b). At 7, 14 and 21 days after Wnt4 overexpression, the expression levels of the three proteins all decreased in the burn wound (Figure 8c). In the epidermis, the three molecules were expressed in the cytoplasm (Figure 8e–g).

## Discussion

Wound closure after burns is one of the most important determinants of survival and long-term outcomes such as functionality and aesthetics [14]. Early wound closure reduces the risk of infection, fluid losses, mortality and length of hospital stay [15]. Closure quality is another critical factor for the prognosis of burn wounds. It determines whether a new skin appendage appears and the degree of hypertrophic scarring. In this study, we found that the overexpression of Wnt4 at the edge of the burn did not change the rate of burn wound closure. Instead, we found that the thickness of the epidermis was increased by Wnt4. Most skin appendages, such as hair follicles, sebaceous glands and sweat glands, consist of epithelium, and hypertrophic scarring is mostly contributed by fibroblasts [16,17]. Thus, we infer that overexpression of Wnt4 may contribute to the healing quality of burn wounds. However, the healing process in mice is not the same as that in humans. Indeed, the lack of an appropriate scarring model has restricted research on healing quality. This conclusion should be validated in human samples in the future.

There are two kinds of Wnt signalling pathways: the canonical Wnt signalling pathway and the noncanonical Wnt signalling pathway. The translocation of β-catenin from the cytoplasm to the nucleus is a sign of the activation of the canonical Wnt signalling pathway [18,19]. β-Catenin binds with Tcf/Lef and activates the transcription of downstream molecules such as cyclinD1 [20]. The noncanonical Wnt signalling pathway consists of several pathways, including the Wnt/Ca^2+^, Wnt/PCP and Wnt/Ror2 signalling pathways [21]. How Wnt4 functions in burn wound healing has not been well studied. In this research, we found that the overexpression of Wnt4 resulted in a decrease in p-β-catenin and an increase in the nuclear translocation of β-catenin. We also found that Wnt4 can bind with Frizzled2 in HaCaT cells and in burn wound skin. Thus, we conclude that Wnt4 activates the canonical Wnt signalling pathway. In addition to activating canonical Wnt signalling, β-catenin also functions in cell adhesion [22]. It can bind with E-cadherin and the cytoskeleton and thus forms part of the anchoring junctions between cells [23]. The role of β-catenin in skin wound healing is complex. It is also involved in other cell activities, such as epithelial–mesenchymal transition [24]. There is considerable controversy as to whether the β-catenin signalling pathway plays a positive role in skin repair, especially for keratinocytes. In some cases, β-catenin can inhibit the migration of cells and promote wound healing [25–27]. In other cases, β-catenin can promote the scarring process [28]. In this study, we found that the expression of β-catenin in the cytoplasm and cell membrane was increased by the overexpression of Wnt4. The secretion of collagen was not impacted. Thus, Wnt4 overexpression-activation of the β-catenin signalling pathway plays a positive role in burn wound healing, though other pathways may also be involved during the burn wound healing process.

When a burn wound occurs, the normal adjacent epidermal cells proliferate and migrate to the wound area [29]. In this study, we found that the proliferation and migration of HaCaT cells were inhibited by the knockdown of Wnt4. This finding is consistent with the functions of Wnt4 in other systems. For example, Wnt4/β-catenin signalling regulates the proliferation and migration of vascular smooth muscle cells [30]. Wnt4 coordinates directional cell migration and extension of the Müllerian duct [31]. Endothelial and steroidogenic cell migration is regulated by Wnt4 in the developing mammalian gonad [32]. Thus, Wnt4 is a positive regulatory factor for the proliferation and migration of epidermal cells. This may be a reason why the overexpression of Wnt4 *in vivo* increased the thickness of epidermal cells. Wnt ligands are glycosylated secretory proteins. Based on our previous study and the literature, it is difficult to purify and maintain their activity after purification [33,34]. In addition, Wnt4 was expressed in HaCaT cells at a high level. These may be the reasons why the use of Wnt4 protein did not promote the proliferation of HaCaT cells. Based on this and our previous study [11], we used an adenoviral gene expression system to overexpress Wnt4 in animals.

**Figure 9:**
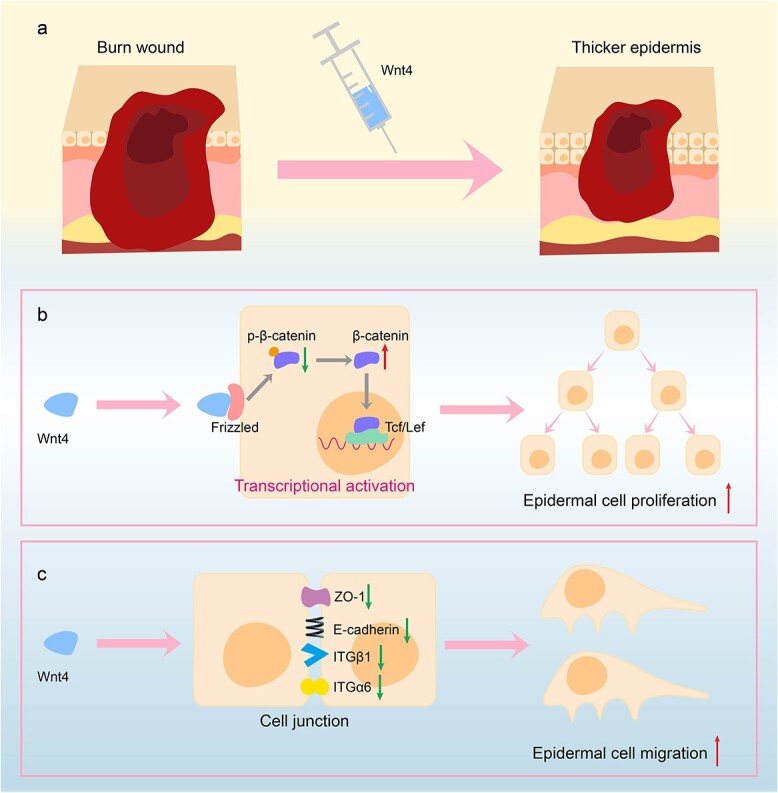
Proposed model for the function of Wnt4 in burn wound healing. (**a**) Wnt4 is overexpressed in burn wounds. (**b**) Wnt4 binds with receptors and abrogates the phosphorylation and degradation of β-catenin. The accumulated β-catenin translocates into the nucleus and transcriptionally activates cell proliferation-related genes, thus promoting the proliferation of epidermal cells. (**c**) The overexpression of Wnt4 results in a decrease in the expression of the cell junction-related proteins ZO-1, E-cadherin, ITGα6 and ITGβ1, thus facilitating the migration of epidermal cells. The increase in the migration and proliferation of epidermal cells together leads to a thicker epidermis during burn wound healing. *ZO-1* Tight junction protein 1, *Tcf* transcription factor, *Lef* lymphoide enhancer binding factor *ITGα6* integrin subunit alpha 6, *ITGβ1* integrin subunit beta 1

Through RNA sequencing, we found that the cell junction-related pathways were among those most impacted when Wnt4 was knocked down in HaCaT cells. Cell proliferation and cell migration depend on cell junctions. Tight junctions are a type of cell junction that function as a barrier for special tissues, such as skin, bone and mucosa. This further supports the conclusion that Wnt4 promotes the proliferation and migration of epidermal cells. Claudin-1, occludin and ZO-1 are widely-used markers for the evaluation of tight junctions [35]. In other systems, all three proteins were mostly expressed at the edge of cells. It is interesting that occludin was mostly expressed in the nucleus of the cells in both the AdWnt4-treated group and the AdGFP-treated group. In addition, the expression of occludin was increased but not decreased by the overexpression of Wnt4. This conflicting result has been reported previously in leaky HK-2 cells [36]. The phenotype of mice lacking occludin varies in different tight-junction-related organs [37]. For example, occludin regulates milk secretion in mammary glands apart from forming tight junctions [38]. This implies that occludin may play roles other than in tight junctions in skin epidermal cells. What functions occludin performs in skin remains to be investigated in future research. Integrins and cadherins are important cell adhesion molecules involved in cell junctions in skin. We examined the expression of ITGα6, ITGβ1 and E-cadherin in AdWnt4-treated burn wound skin. Consistent with our expectations, the expression of the three cell adhesion molecules was decreased by AdWnt4. E-Cadherin is closely related to cell migration during wound healing [39]. ITGα6 was reported to be a positive factor for wound healing [40,41]. The role of ITGβ1 in wound healing is controversial [42,43]. Burn wound healing is a complex process and not all the results are consistent with the literature. This may be due to the possibility that these cell adhesion molecules may also be involved in other cell activities in addition to cell migration. Based on our results, we propose a work model, which is summarized in Figure 9.

## Conclusions

Our study reports the function of Wnt4 in burn wound healing and the potential mechanisms. Overexpression of Wnt4 increases the expression and nuclear translocation of β-catenin, activates the canonical Wnt signalling pathway and decreases the expression of the cell junction proteins ZO-1, E-cadherin, ITGα6 and ITGβ1, thus increasing the proliferation and migration of epidermal cells. The thicker epidermis may result in a decrease in scarring, thus increasing the healing quality of burn wounds. In summary, our study reveals a new possible method and a new target to increase the healing quality of burn wounds.

## Supplementary Material

Table_S1_Primer_sequences_tkac053

## Data Availability

The datasets used and/or analysed in the current study are available from the corresponding author upon reasonable request.

## Abbreviations

AdGFP: Empty vector for AdWnt4; AdWnt4: Adenovirus-mediated overexpression of mouse Wnt4; DAPI: 4′,6-Diamidino-2-phenylindole; GFP: Green fluorescent protein; H&E: Haematoxyline and eosin; shGFP: Empty vector for shWnt4; shWnt4: shRNA of Wnt4 mediated by lentivirus.
